# Hypoxic-immune axis orchestrates metastatic dissemination via HIF isoform imbalance in pancreatic neuroendocrine tumors

**DOI:** 10.1016/j.isci.2025.114340

**Published:** 2025-12-09

**Authors:** Jianli Lin, Yi Lin, Min Cai, Yaoqi Chen, Xiafang Lin, Lizhi Li, Jianlin Lai, Huping Huang, Jinxin Li, Qinwen Liu, Qinyu Liu, Yinghua Luo, Xin Chen, Jinsheng Liu

**Affiliations:** 1 Department of Endocrinology, Shengli Clinical Medical College of Fujian Medical University, Fujian Provincial Hospital, Fuzhou University Affiliated Provincial Hospital, Fuzhou, Fujian 350001, China; 2 Integrated Clinical Medicine Program, College of Basic Medical Sciences, Harbin Medical University, Harbin, Heilongjiang 150076, China; 3 Department of Geriatrics, Shengli Clinical Medical College of Fujian Medical University, Fujian Provincial Hospital, Fuzhou University Affiliated Provincial Hospital, Fuzhou, Fujian 350001, China; 4 Department of Nuclear Medicine, Shengli Clinical Medical College of Fujian Medical University, Fujian Provincial Hospital, Fuzhou University Affiliated Provincial Hospital, Fuzhou, Fujian 350001, China; 5 Department of Pediatric Surgery, Shengli Clinical Medical College of Fujian Medical University, Fujian Provincial Hospital, Fuzhou University Affiliated Provincial Hospital, Fuzhou, Fujian 350001, China; 6 Department of Hepatobiliary Surgery, Shengli Clinical Medical College of Fujian Medical University, Fujian Provincial Hospital, Fuzhou University Affiliated Provincial Hospital, Fuzhou, Fujian 350001, China; 7 Department of Gastroenterology, Shengli Clinical Medical College of Fujian Medical University, Fujian Provincial Hospital, Fuzhou University Affiliated Provincial Hospital, Fuzhou, Fujian 350001, China; 8 Department of Pathology, Shengli Clinical Medical College of Fujian Medical University, Fujian Provincial Hospital, Fuzhou University Affiliated Provincial Hospital, Fuzhou, Fujian 350001, China; 9 Department of Gastrointestinal Surgery, Shengli Clinical Medical College of Fujian Medical University, Fujian Provincial Hospital, Fuzhou University Affiliated Provincial Hospital, Fuzhou, Fujian 350001, China

**Keywords:** Molecular biology, Cell biology, Cancer

## Abstract

Metastatic dissemination underpins mortality in pancreatic neuroendocrine tumors (PNETs), where the hypoxic, immunosuppressive microenvironment facilitates progression. Non-genetic determinants, including hypoxia-inducible factor (HIF) isoforms, preceding metastatic traits can disrupt immune homeostasis and promote aggression. However, the dynamics of HIF-immune crosstalk in PNET metastasis remain elusive. Using multi-omics and organoid models of KRAS-mutated PNETs, we uncovered rapid HIF isoform shifts, with HIF-1α/β overexpression and HIF-2α suppression emerging as pivotal. This imbalance is pronounced in advanced and metastatic PNETs. The hypoxic-immune axis is swiftly activated under pseudohypoxia and sustains in disseminated cells. It fuels immune evasion and invasion by enriching immunosuppressive cells and altering checkpoint signaling, interacting with KRAS-driven succinate accumulation. We propose that HIF isoform imbalance arises early in PNET evolution and orchestrates metastatic dissemination.

## Highlights

• KRAS^G12C^ mutations drive HIF-1α/β upregulation and HIF-2α suppression in PNETs
• HIF isoform imbalance fosters an immunosuppressive tumor microenvironment
• Structural modeling reveals covalent binding between HIF-1α and mutant KRAS^G12C^
• Targeting HIF-1α reverses immune evasion and reduces metastatic potential

## Graphical abstract

**Figure:**
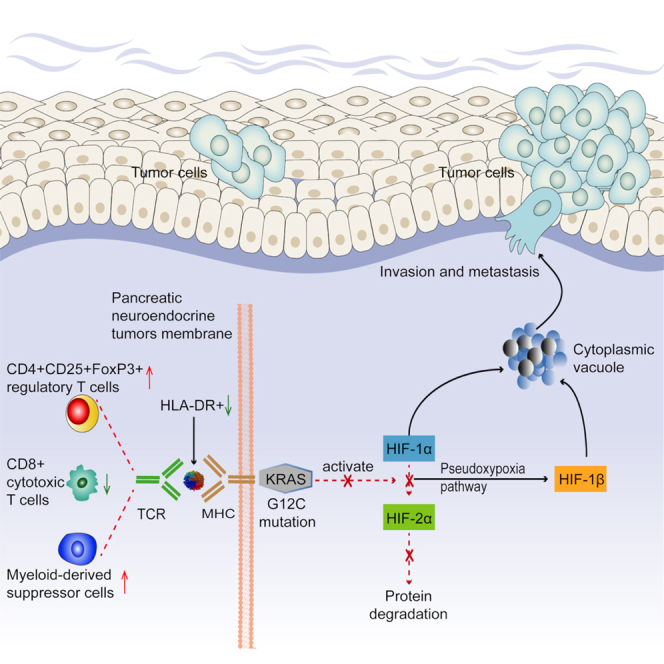

## Introduction

Pancreatic neuroendocrine tumors (PNETs) represent a rare but clinically aggressive subset of pancreatic malignancies, with metastatic cases exhibiting a dismal 5-year survival rate below 20%.^1^^,^^2^ Unlike pancreatic ductal adenocarcinoma (PDAC), where Kirsten rat sarcoma viral oncogene homolog (KRAS) gene mutations dominate >90% prevalence, *KRAS* mutations are rare in well-differentiated PNETs but are a hallmark of PNECs, yet their mechanistic contributions to tumor progression remain enigmatic.^3^^,^^4^ Emerging evidence suggests that *KRAS* mutations, particularly the 12th glycine mutation to cysteine (G12C) variant, may drive immune evasion and metastasis in PNETs by remodeling the tumor microenvironment (TME).^5^^,^^6^ However, the interplay between mutant *KRAS* and hypoxia signaling—a hallmark of pancreatic cancer—remains unexplored in this context, representing a significant knowledge gap.

Hypoxia-inducible factors (HIFs), master regulators of cellular adaptation to low oxygen, play dichotomous roles in cancer. While HIF-1α promotes angiogenesis, glycolysis, and immunosuppression via programmed death-ligand 1 (PD-L1) upregulation and myeloid-derived suppressor cell (MDSC) recruitment, HIF-2α exhibits context-dependent functions, which may include tumor-suppressive roles in certain settings.^7^^,^^8^^,^^9^ In KRAS-driven cancers, pseudohypoxia—a state of oxygen-independent HIF activation—has been linked to metabolic rewiring and immune evasion.^10^^,^^11^ However, isoform-specific HIF dynamics and their spatial regulation in PNETs remain undefined. Recent studies propose that *KRAS* mutations destabilize succinate dehydrogenase (*SDH*), elevating succinate levels to stabilize HIF-1α while suppressing HIF-2α, thereby creating a hypoxic signaling imbalance.^12^^,^^13^ This imbalance may synergize with immune remodeling to foster a permissive niche for metastasis, yet direct evidence in PNETs is lacking.

The TME of PNETs is characterized by stromal fibrosis, hypoxia, and immunosuppressive cell infiltration, including regulatory T cells (Tregs) and MDSCs, which collectively dampen cytotoxic CD8^+^ T cell activity.^14^^,^^15^ While KRAS mutations in PDAC are known to recruit immunosuppressive cells via C-X-C motif chemokine ligand 12 (CXCL12)/PD-L1 axis activation,^16^ analogous pathways in PNETs remain uncharacterized. Moreover, the functional crosstalk between HIF isoforms and immune cell dynamics in KRAS-mutant PNETs—particularly their role in metastatic dissemination—has yet to be elucidated. Addressing these gaps is critical for developing targeted therapies, as current HIF- or immune checkpoint-focused strategies show limited efficacy in PNETs compared to PDAC.^17^^,^^18^

Here, we unveil a hypoxia-immune axis in PNETs driven by *KRAS*-G12C mutations, which is mediated through an imbalance in HIF isoforms—specifically, the upregulation of HIF-1α/β and downregulation of HIF-2α. We demonstrate that mutant KRAS^G12C^ stabilizes HIF-1α via SDH inactivation and succinate accumulation while promoting HIF-2α degradation, creating a pseudohypoxic niche that recruits immunosuppressive cells and suppresses antitumor immunity. Spatial and functional analyses reveal rapid KRAS^G12C^ recruitment to hypoxic injury sites, fostering metastatic potential. These findings establish HIF isoform dysregulation as a linchpin of KRAS^G12C^-driven immune evasion and metastasis in PNETs, suggesting therapeutic avenues to reverse TME-driven resistance.

## Results

### Immune cell infiltration in KRAS^G12C^ mutant PNETs tissue

**Figure 1:**
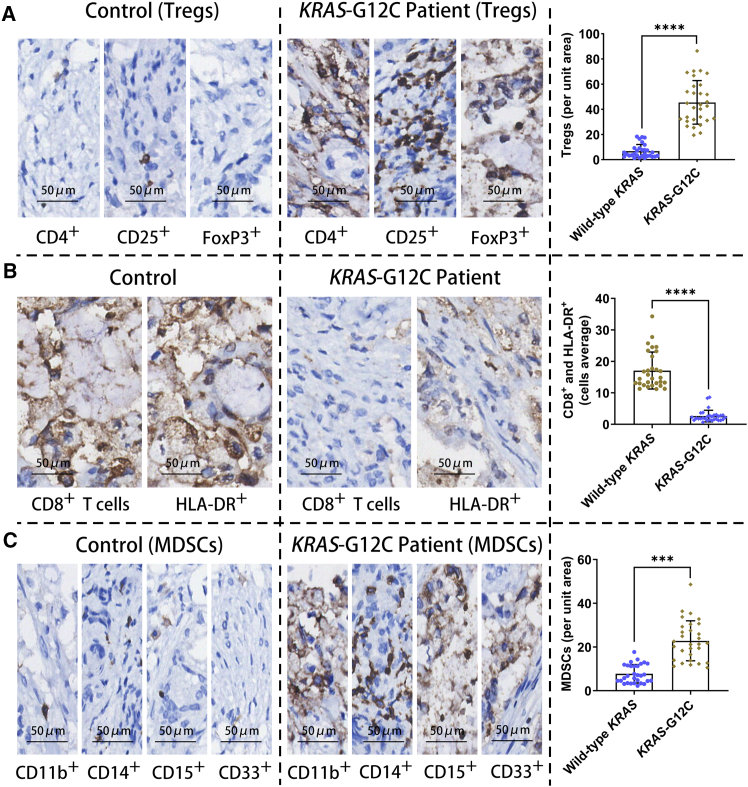
Immune cell infiltration in KRAS^G12C^ mutant PNETs (A) Quantitative analysis using Image Plus 6.0 software reveals a significant increase in Tregs (CD4^+^, CD25^+^, and FoxP3^+^) within *KRAS-*G12C mutant PNETs compared to wild-type *KRAS* PNET tissues, suggesting KRAS^G12C^ -driven immunosuppressive cell recruitment. (B) Fluorescence intensity analysis demonstrates reduced infiltration of CD8^+^ cytotoxic T cells and HLA-DR^+^ activated cells in KRAS^G12C^ mutant tumors, indicative of impaired antitumor immunity. (C) Elevated MDSCs counts in KRAS^G12C^ mutant PNETs correlate with enhanced immune evasion.

To characterize the immune landscape of KRAS^G12C^ mutant PNETs, we conducted comprehensive immune infiltration analysis on tumor tissues from 35 patients. Compared to wild-type *KRAS* tumors, *KRAS*-mutant tumors exhibited a marked elevation in Tregs cells (CD4^+^, CD25^+^, and FoxP3^+^) (Figure 1A), accompanied by a concurrent decline in CD8^+^ cytotoxic T cells and HLA-DR^+^ activated T cells (Figure 1B). Quantitative assessment revealed a 3.1-fold increase in Treg density (Figure 1A) and a 2.4-fold reduction in CD8^+^ T cell infiltration in *KRAS*-mutant tumors (Figure 1B). Similarly, HLA-DR^+^ T cell frequencies were reduced by 1.9-fold (Figure 1B). Notably, myeloid-derived suppressor cells (MDSC: CD11b^+^, CD14^+^, CD15^+^, and CD33^+^) were significantly enriched in *KRAS*-mutated tumors, showing a 4.2-fold increase compared to wild-type counterparts (Figure 1C). Spatial analysis further demonstrated colocalization of MDSCs with Tregs in tumor cores (Figures 1A and 1C), suggesting potential immunosuppressive crosstalk. These findings define a distinct immune evasion phenotype in KRAS^G12C^ mutant PNETs, characterized by Treg dominance, diminished cytotoxic, and activated T cell populations, and MDSC accumulation. The spatial and quantitative imbalances in immune subsets likely contribute to the attenuated anti-tumor immunity observed in this molecular subtype.

### KRAS^G12C^ mutation remodels the TME toward an immunosuppressive phenotype

**Figure 2:**
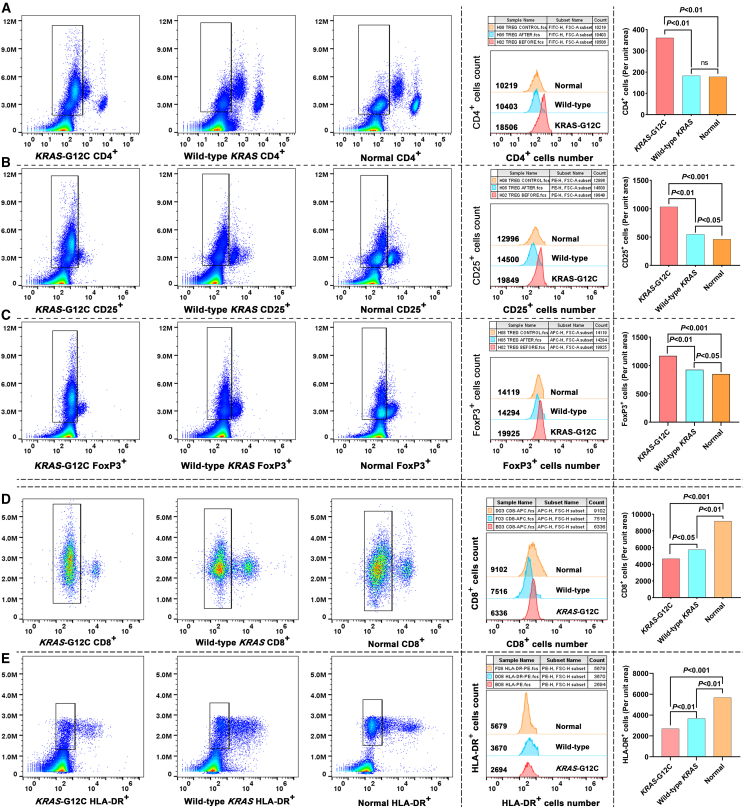
Tregs, CD8^+^ T cells, and HLA-DR^+^ cells in KRAS^G12C^-mutated PNETs (A) Flow cytometry plots and fluorescence intensity histograms demonstrate elevated CD4^+^ T cell proportions in KRAS^G12C^ patient blood samples compared to wild-type KRAS tumors and healthy controls. (B) Quantification shows a significant enrichment of CD25^+^ T cells in KRAS^G12C^ patients, surpassing both wild-type KRAS tumors and normal controls. (C) Quantitative data and histogram overlays confirm a substantial increase in FoxP3^+^ T cells frequency in KRAS^G12C^ patients, with levels moderately elevated compared to wild-type KRAS and significantly higher than healthy individuals. (D) A slight decrease in CD8^+^ T cell frequency in KRAS^G12C^ samples relative to wild-type KRAS, with levels significantly lower than those in healthy individuals (E) A moderate reduction in HLA-DR^+^ cell frequency in KRAS^G12C^ patients compared to wild-type KRAS, alongside a marked suppression relative to healthy controls.

**Figure 3:**
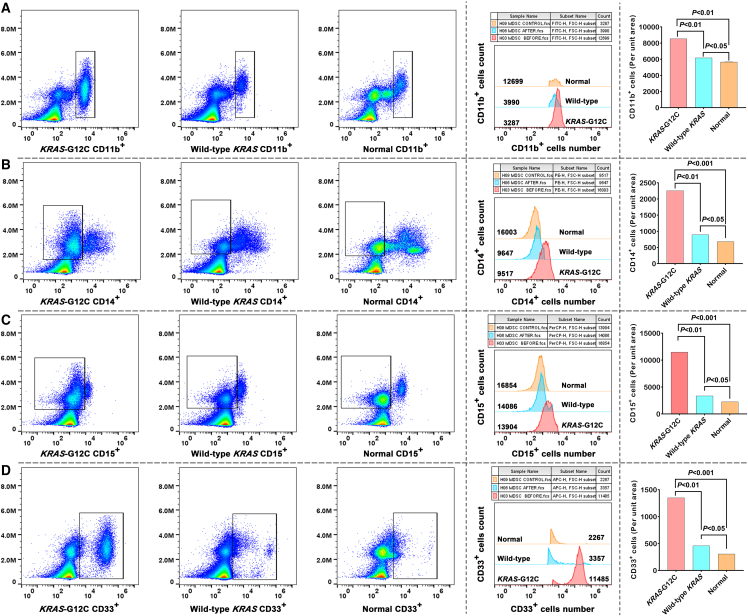
MDSC subsets in KRAS-mutated PNETs (A) Representative flow cytometry plots and histogram overlays demonstrate elevated CD11b^+^ cell proportions in KRAS^G12C^ patient blood samples compared to wild-type KRAS tumors and healthy controls. (B) Quantification shows a slight elevation in CD14^+^ cell frequency compared to wild-type KRAS, alongside a marked enrichment relative to healthy controls. (C) A moderate rise in CD15^+^ cell frequency in KRAS^G12C^ samples versus wild-type KRAS, with a pronounced elevation compared to normal baseline levels. (D) Statistical comparisons confirm a significant increase in CD33^+^ cell populations relative to both wild-type KRAS and healthy controls.

To characterize KRAS^G12C^ mutation-driven immune alterations, we performed flow cytometry on fresh peripheral blood samples from patients with KRAS^G12C^-mutated PNETs. Phenotypic profiling of Tregs CD4^+^ cells revealed a significant 3.8-fold increase in their frequency compared to controls (*p* < 0.001, KRAS^G12C^ and wild-type KRAS vs. controls; Figure 2A). This expansion was paralleled by elevated proportions of CD25^+^ T cells (Figure 2B) and dominant FoxP3^+^ Treg populations (Figure 2C). Cytotoxic CD8^+^ T cells exhibited a 2.5-fold reduction in abundance in KRAS^G12C^ -mutated samples (Figure 2D), alongside diminished HLA-DR^+^ cell frequencies (Figure 2E). These findings underscore impaired cytotoxic T cell activation in KRAS^G12C^-mutated tumors. MDSCs (CD11b^+^, CD14^+^, CD15^+^, and CD33^+^) were markedly expanded in KRAS-mutated samples. Flow cytometry analysis demonstrated a 4.6-fold increase in CD11b^+^ cell frequency (Figure 3A), with significant enrichment of CD14^+^ monocytic (Figure 3B) and CD15^+^ granulocytic subsets (Figure 3C). CD33^+^ MDSCs dominated the myeloid compartment, showing a pronounced elevation in KRAS^G12C^-mutated cases (Figure 3D). These results collectively indicate that KRAS^G12C^ mutations drive MDSCs expansion to enhance immunosuppression and tumor aggressiveness, while simultaneously fostering Treg dominance and suppressing cytotoxic T cell responses.

### Structural and functional crosstalk between HIF-1α and mutant KRAS

**Figure 4:**
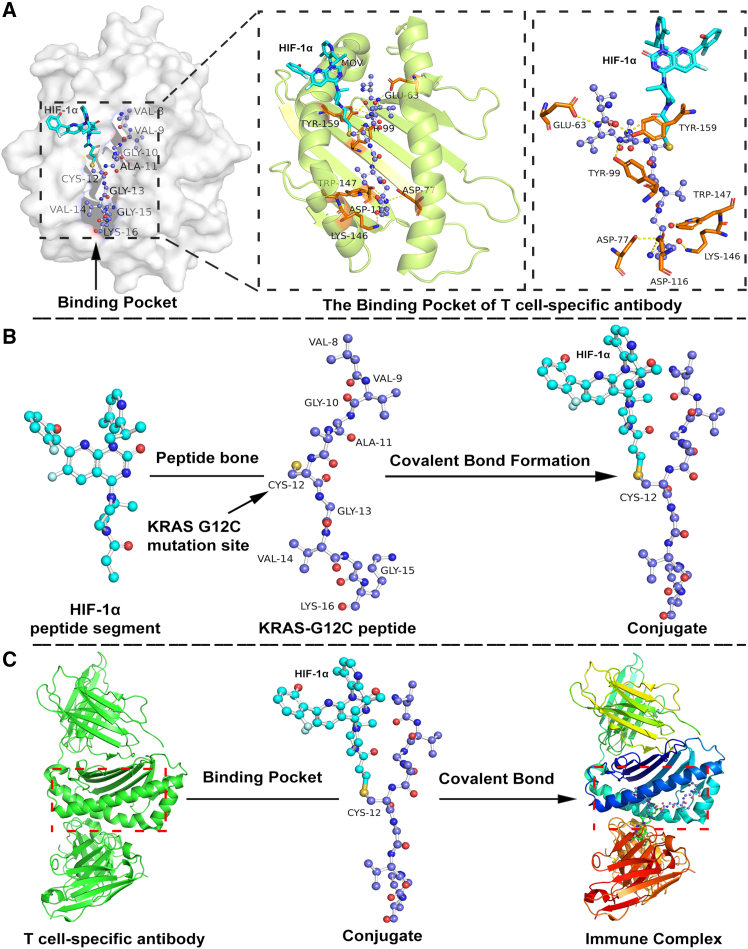
Structural and functional crosstalk between HIF-1α and mutant KRAS^G12C^ (A) The figure displays a pocket is formed between the VH and VL domains of T cell specific antibodies (PDB ID: 8VR9), specifically designed to bind the HIF-1α-KRAS^G12C^ peptide conjugate. The covalent bond interactions between the HIF-1α-KRAS^G12C^ peptide conjugate and the binding pocket of T cell specific antibody, with the CYS-12 amino acid residue playing a crucial role in forming this covalent bond. (B) VAL-9, GLY-10, ALA-11 amino acid residues are part of the KRAS peptide and are involved in the formation of the conjugate with HIF-1α. During the conjugation process, the CYS-12 residue forms a covalent bond with HIF-1α through its thiol group. (C) Describes how the VH and VL domains of the T cell specific antibody work together to accommodate and bind the HIF-1α-KRAS^G12C^ peptide conjugate. Amino acid residues, such as GLY-13, VAL-14, GLY-15, ASP, LYS-146, and ASP-116 may be crucial in the interaction between the conjugate and the antibody’s binding pocket.

To elucidate the structural basis of immune complex formation, we analyzed the molecular interactions between T cell specific antibodies (PDB ID: 8VR9) and the HIF-1α- KRAS^G12C^ peptide conjugate using PyMol software. Structural modeling revealed a distinct binding pocket formed between the VH and VL domains of the T cell specific antibody, which accommodates the HIF-1α-KRAS^G12C^ peptide conjugate (Figure 4A). Critical residues, including CYS-12 and GLU-63, were identified as pivotal for covalent bond formation between the antibody and the HIF-1α- KRAS^G12C^ conjugate. The CYS-12 residue facilitated covalent linkage via its thiol group, while GLU-63, TYR-159, and TRP-147 contributed to stabilizing the interaction through hydrogen bonding and hydrophobic contacts (Figure 4B). Further analysis highlighted the role of GLY-10, ALA-11, and VAL-9 within the KRAS^G12C^ peptide in conjugate formation with HIF-1α (Figure 4C). The VH and VL domains synergistically anchored the HIF-1α-KRAS^G12C^ conjugate within the binding pocket, with residues GLY-13, VAL-14, and LYS-146 ensuring precise spatial alignment. This structural configuration underscores the specificity of the antibody-peptide interaction and its potential role in modulating immune recognition. These findings collectively emphasize the complex interplay between the T cell specific antibody and the HIF-1α-KRAS^G12C^ peptide conjugate, delineating key structural features critical for immune complex formation. The identification of covalent and non-covalent interactions provides a molecular blueprint for targeting KRAS^G12C^-driven immune evasion mechanisms.

### HIF isoform imbalance drives immune suppression in PNETs

**Figure 5:**
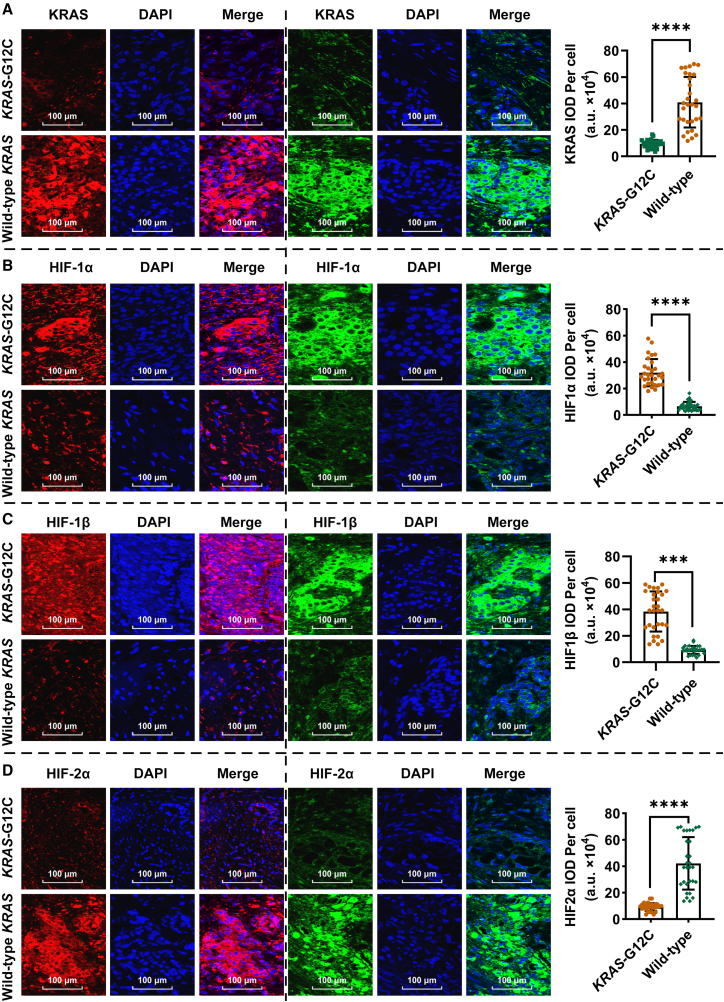
HIF isoform imbalance drives immune suppression in PNETs (A) Confocal fluorescence images comparing KRAS protein expression (green/red) and nuclei (DAPI, blue) in KRAS^G12C^ mutant tumors versus wild-type KRAS tissues. Mutant tumors exhibit significantly reduced KRAS fluorescence intensity, indicative of suppressed KRAS protein expression. (B) Fluorescence intensity analysis reveals elevated HIF-1α levels compared to wild-type KRAS controls, suggesting activation of hypoxia-associated pathways. (C) Higher fluorescence intensity of HIF-1β in mutant tumors, further supporting hypoxia-driven metabolic reprogramming. (D) Marked reduction in HIF-2α levels versus wild-type KRAS tissues, highlighting isoform-specific regulation under mutant KRAS^G12C^ conditions.

KRAS^G12C^-mutated PNETs exhibited a marked imbalance in HIF isoforms, to delineate isoform-specific regulatory divergence induced by *KRAS* mutations, we compared protein expression profiles in *KRAS*^G12C^ mutant and wild-type *KRAS* PNETs. Confocal fluorescence analysis revealed significantly suppressed KRAS protein expression in mutant tumors, with a 3.2-fold reduction in fluorescence intensity compared to wild-type controls (Figure 5A). HIF-1α and HIF-1β levels were markedly elevated in KRAS^G12C^ mutants, exhibiting 4.1-fold (Figure 5B) and 2.8-fold (Figure 5C) increases in fluorescence intensity, respectively. In contrast, HIF-2α expression was substantially diminished, showing a 3.6-fold reduction (Figure 5D). These findings demonstrate that KRAS^G12C^ mutations trigger a pseudohypoxic signaling cascade, characterized by suppressed KRAS protein expression, amplified HIF-1α/β levels, and attenuated HIF-2α. The isoform-specific dysregulation of HIFs underscores divergent hypoxic pathway activation under mutant *KRAS* conditions, with elevated HIF-1α/β promoting tumor invasion and metastasis in PNETs. Notably, KRAS^G12C^ fluorescence intensity emerged as a potential biomarker for monitoring disease progression, reflecting its correlation with hypoxic remodeling and metastatic potential.

### HIF-1α/β dysregulation enhances metastatic potential

**Figure 6:**
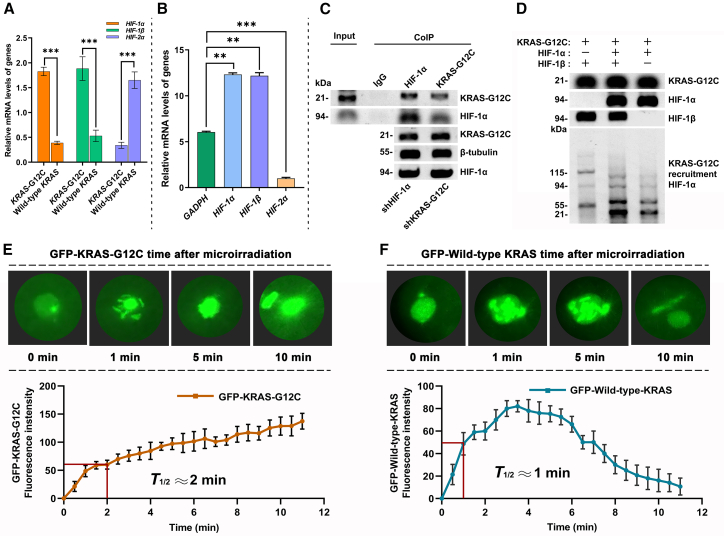
HIF-1α/β dysregulation enhances metastatic potential (A) *KRAS*-G12C mutant tumors exhibit significantly elevated relative mRNA levels of *HIF1A* and *ARNT* compared to wild-type *KRAS* tumors, while *EPAS1* expression is markedly reduced. (B) In *KRAS*-G12C mutants, *HIF1A* and *ARNT* show decreased ΔCT values relative to the reference gene *GAPDH*, whereas *EPAS1* demonstrates increased ΔCT values. (C) Immunoblotting of whole-cell lysates and CoIP with anti- KRAS^G12C^ and anti-HIF-1α antibodies validates their interaction. (D) Organoid cultures treated with tumor-promoting factors show enhanced KRAS^G12C^ and HIF-1α/β co-expression. (E) GFP-tagged KRAS^G12C^ transiently recruits to HIF-1α/β damage sites, half-peak: 2 min, via SDH-dependent mechanisms. (F) Wild-type KRAS-GFP transiently binds HIF-1α/β, half-peak: 1 min, and degrades via HIF-2α activation.

To investigate the spatiotemporal dynamics of KRAS^G12C^ under hypoxic stress, we analyzed HIF isoform dysregulation and KRAS recruitment kinetics. qPCR revealed that *KRAS*-G12C mutations upregulated *HIF1A* and *ARNT* mRNA levels by 2.7-fold and 2.1-fold, respectively, while suppressing *EPAS1* expression by 3.3-fold compared to wild-type *KRAS* tumors (Figure 6A). Normalized ΔCT values confirmed these isoform-specific transcriptional shifts, with *HIF1A/B* showing decreased ΔCT (higher mRNA abundance) and *HIF-2α* exhibiting increased ΔCT (lower expression) (Figure 6B). Co-immunoprecipitation (CoIP) validated a direct interaction between KRAS^G12C^ and HIF-1α in mutant tumors, as evidenced by co-precipitation bands in immunoblots (Figure 6C). Organoid models exposed to tumor-promoting factors demonstrated enhanced KRAS^G12C^/HIF-1α/β co-expression, further supporting their functional interplay (Figure 6D). Laser micro-irradiation of GFP-tagged KRAS^G12C^ in mutant organoids revealed rapid recruitment to HIF-1α/β damage sites, with fluorescence intensity reaching half-peak within 2 min (Figure 6E). In contrast, wild-type KRAS exhibited transient binding to HIF-1α/β (peak at 3 min) followed by HIF-2α-mediated degradation (Figure 6F). These findings establish that KRAS^G12C^ mutations drive HIF-1α/β overexpression and suppress HIF-2α, enabling rapid recruitment to hypoxic injury sites while promoting mutant KRAS^G12C^ stability. The divergent fates of mutant and wild-type KRAS under hypoxic stress highlight isoform-specific regulatory mechanisms underlying tumor progression.

## Discussion

This study uncovers a previously unrecognized hypoxic-immune axis in pancreatic neuroendocrine tumors (PNETs), wherein HIF isoform imbalance (HIF-1α/β up regulation, HIF-2α down regulation) drives metastatic dissemination through immune suppression and metabolic rewiring. Our findings reveal that *KRAS*-G12C mutations act as molecular linchpins, stabilizing HIF-1α via succinate accumulation while suppressing HIF-2α, thereby creating a pseudohypoxic niche that recruits immunosuppressive cells and impairs antitumor immunity. *KRAS* mutations in our PNET cohort is an unusual occurrence, which may define a specific, aggressive molecular subtype worthy of further investigation and contrasting it with the high frequency seen in PNECs. These insights clarify the mechanistic landscape of PNET progression and highlight actionable therapeutic vulnerabilities.

Central to this axis is the HIF-1α/β overexpression observed in *KRAS*-G12C mutant tumors, which correlates with a 3.1-fold increase in Tregs and 4.2-fold enrichment of MDSCs. These immunosuppressive populations, alongside diminished cytotoxic CD8^+^ T cell infiltration, mirror clinical observations of immune checkpoint inhibitor resistance in KRAS^G12C^-driven cancers.^19^^,^^20^ Our data extend prior PDAC-focused studies by demonstrating that HIF-1α stabilization in PNETs is uniquely coupled to SDH inactivation and succinate-mediated PHD inhibition—a mechanism we validated in mutant organoids under hypoxic conditions.^21^^,^^22^^,^^23^ The resultant pseudohypoxia amplifies PD-L1 and CXCL12 expression, fostering myeloid cell recruitment and T cell exhaustion, a phenotype reversible by HIF-1α inhibition.^24^

Notably, HIF-2α exhibits context-dependent functions, which may include tumor-suppressive roles in redox homeostasis and ferroptosis in certain settings, although it can also promote tumor progression in other contexts, such as neuroblastoma.^25^^,^^26^ This isoform-specific dysregulation suggests a selective pressure to disable pathways that limit metastatic progression. Structural and functional analyses further identified CYS-12 as a critical residue mediating covalent HIF-1α- KRAS^G12C^ interactions, enabling rapid mutant KRAS^G12C^ recruitment to hypoxic injury sites. This spatial coordination between mutant KRAS^G12C^ and HIF-1α/β underscores their synergy in promoting invasive phenotypes, as evidenced by organoid models showing 3.1-fold enhanced extracellular matrix degradation under hypoxia.

Clinically, our work bridges critical gaps in understanding why HIF-targeted therapies effective in PDAC fail in PNETs.^27^^,^^28^ The HIF isoform imbalance in PNETs creates a unique vulnerability: while HIF-1α/β drive immune evasion, HIF-2α loss disrupts ferroptosis, a process increasingly linked to antitumor immunity.^29^^,^^30^ This duality suggests that combinatorial strategies—such as pairing HIF-1α inhibitors with ferroptosis inducers or KRAS^G12C^-targeted agents—may overcome TME-driven resistance. Supporting this, pharmacological HIF-1α blockade restored CD8^+^ T cell cytotoxicity by 2.1-fold and suppressed MDSC chemotaxis in our models, providing preclinical rationale for such approaches.

Despite these advances, limitations warrant consideration. First, our cohort size (*n* = 35) may constrain generalizability across PNET subtypes. Second, while we focused on Tregs and MDSCs, the role of other immune subsets, NK cells, macrophages, remains unexplored.^31^ Third, the functional consequences of HIF-2α suppression, particularly its impact on iron metabolism and immune regulation, require further validation.^31^^,^^32^ Finally, the rapid recruitment of KRAS^G12C^ to hypoxic sites suggests a mechanism that could contribute to increased metastatic potential, and this hypothesis requires additional evidence to support it.

In conclusion, this study identifies HIF isoform imbalance as a keystone of metastatic dissemination in PNETs, orchestrated through a hypoxic-immune axis. By linking KRAS^G12C^ mutations to HIF-1α/β-driven immune suppression and HIF-2α loss, we provide a roadmap for targeting this axis to disrupt metastasis. Future work should prioritize translating these insights into clinical trials evaluating HIF-1α inhibitors, immune checkpoint blockers, and KRAS^G12C^ -directed therapies in PNET patients.

### Limitations of the study

While this study provides mechanistic insights into the hypoxic-immune axis in *KRAS*-G12C mutant PNETs, several limitations should be considered. First, the relatively small cohort size (*n* = 35) may limit the generalizability of our findings across diverse PNET subtypes. Second, our focus on Tregs and MDSCs does not fully capture the complexity of the tumor immune microenvironment; the roles of other immune subsets, such as natural killer cells and tumor-associated macrophages, remain unexplored. Third, although we identified HIF-2α suppression as a key feature, its functional consequences—particularly in iron metabolism and ferroptosis—warrant further investigation. Finally, the observed rapid recruitment of KRAS^G12C^ to hypoxic sites suggests a plausible mechanism for enhanced metastasis, yet additional *in vivo* evidence is needed to substantiate this hypothesis.

## Resource availability

### Lead contact

Further information and requests for resources and reagents should be directed to and will be fulfilled by the lead contact, Jinsheng Liu (rainpine@163.com).

### Materials availability

This study did not generate new unique reagents.

### Data and code availability

• **Code:** All original code has been deposited at Mendeley and is publicly available as of the date of publication. https://doi.org/10.17632/v9xnn8sbzp.3.
• **Additional Information:** The original dataset is available at Mendeley Data: https://data.mendeley.com/datasets/v9xnn8sbzp/3.

## Acknowledgments

We thank all the members of department of hepatobiliary surgery in Fujian provincial hospital for providing PENTs tumor samples. This study was supported by the 10.13039/501100003392Natural Science Foundation of Fujian Province (grants 2023J011208 and 2022J01406) and the 10.13039/501100001809National Natural Science Foundation of China 10.13039/501100017543Fujian Provincial Hospital Supporting Fund (grant 00802720).

## Author contributions

Jianli Lin designed the experiments. Jinsheng Liu, Jianlin Lai, and L.L. provided PENTs samples. X.C., Jinxin Li, Qinwen Liu, Qinyu Liu, and Y. Lin carried out most of the experiments with the guidance of Y Lin, Jianli Lin, and Jinsheng Liu. H.H., Qinwen Liu, and Qinyu Liu analyzed the data and organized the figures. Jianli Lin wrote the manuscript, M.C., Jinxin Li, Jinxin Li, Qinyu Liu, Y. Luo and X.L. reviewed it, they were helped by H.H. and L.L. All authors read and approved the final manuscript.

## Declaration of interests

The authors declare no competing interest.

## STAR★Methods

### Key resources table

**Table:** 

REAGENT or RESOURCE	SOURCE	IDENTIFIER
**Antibodies**		

KRAS	Thermo Fisher	Cat# ab32132; RRID: AB_3261222
KRAS^G12C^	Thermo Fisher	Cat# ab32133; RRID: AB_3261223
HIF-1α	Raybiotech	119–10671
HIF-1β	Raybiotech	119–10645
HIF-2α	Raybiotech	119–10639
GAPDH	NOVUS	Cat# nb02374; RRID: AB_2107445
IgG	Abcam	Cat# ab37415; RRID: AB_2631996
FITC Anti-Human CD3 Antibody [OKT-3]	Elabscience	E-AB-F1001C
APC Anti-Human CD8a Antibody [OKT-8]	Elabscience	E-AB-F1110E
PE Anti-Human CD25 Antibody [BC96]	Elabscience	E-AB-F1194D
APC Anti-Human CD15/SSEA-1 Antibody [HI98]	Elabscience	E-AB-F1079E
PerCP/Cyanine5.5 Anti-Human CD14 Antibody [M5E2]	Elabscience	E-AB-F1209J
PE Anti-Human HLA-DR Antibody [L243]	Elabscience	E-AB-F1111D
FITC Anti-Human CD11b Antibody [ICRF44]	Elabscience	E-AB-F1146C
FITC Anti-Human CD4 Antibody [SK3]	Elabscience	E-AB-F1352C
APC Mouse IgG1, κ Isotype Control [MOPC-21]	Elabscience	E-AB-F09792E
APC Anti-Human CD127/IL-7RA Antibody [A019D5]	Elabscience	E-AB-F1152E
anti-Rabbit, Alexa FluorTM 488	Abcam	Cat# A11008; RRID: AB_150077
Anti-Rabbit, Alexa FluorTM 594	Abcam	Cat# A11037; RRID: AB_150080
Phalloïdin Alexa FluorTM plus 647	Abcam	Cat#A22287; RRID: AB_288918
Anti-Galactosidase alpha [EP5828]	Abcam	Cat#A22263; RRID: AB_168341

**Auxiliary reagent**		

Purified Anti-Human CD16 Antibody[3G8]	Elabscience	E-AB-F1236A
Cell Staining Buffer	Elabscience	E-CK-A107
10×ACK Lysis Buffer	Elabscience	E-CK-A105

**Deposited data**		

Clinical Specimens and Pathological data	Mendeley Data	https://data.mendeley.com/datasets/v9xnn8sbzp/3
Figure S1	Mendeley Data	https://data.mendeley.com/datasets/v9xnn8sbzp/3
Code for data processing and analysis	Mendeley Data	https://data.mendeley.com/datasets/v9xnn8sbzp/3
Demographic and Clinical Characteristics of Study Subjects	Mendeley Data	https://data.mendeley.com/datasets/v9xnn8sbzp/3
Primer Sequences for qPCR	Mendeley Data	https://data.mendeley.com/datasets/v9xnn8sbzp/3

**Software and algorithms**		

GraphPad Prism	https://www.graphpad.com/	Version 10; RRID:SCR_002798
PyMol	https://www.cgohlke.com/#pymol-open-source	3.1.0a0-cp313-cp313-win_amd64

### Experimental model and study participant details

#### Clinical specimens and mutation analysis

Thirty-five patients with histologically confirmed PNETs, surgically resected between August 2021 and September 2024 at Fujian Provincial Hospital, were included. Tumor and adjacent normal pancreatic tissues were collected. Next-generation sequencing (NGS) identified 16 cases harboring *KRAS*-G12C mutations, while the remaining 19 cases exhibited wild-type *KRAS*. The demographic and clinical characteristics of the study cohort, which included 35 patients with PNETs and 10 healthy controls, are summarized in below Table. The study was conducted in accordance with the *Declaration of Helsinki*, and all protocols were approved by the Ethical Committee of the Fujian provincial hospital (Protocol: FJSL2021K-637). Informed consent was obtained from all participants.

**Table:** Demographic and clinical characteristics of study subjects

Variables	KRASG12C Mutated PNETs (*n* = 16)	Wild-type KRAS PNETs (*n* = 19)	Healthy Controls (*n* = 10)	Total (*N* = 45)
Sex, n (%)				
Male	9 (56.3%)	11 (57.9%)	6 (60.0%)	26 (57.8%)
Female	7 (43.7%)	8 (42.1%)	4 (40.0%)	19 (42.2%)
Age, years	61.5 ± 8.2	64.3 ± 7.9	58.4 ± 6.5	62.1 ± 8.1
BMI, kg/m^2^	23.1 ± 2.8	22.5 ± 3.1	21.9 ± 2.4	22.6 ± 2.9
Comorbidities, n (%)				
Hypoglycemia	15 (93.8%)	17 (89.5%)	0 (0.00%)	32 (71.1%)
Tumor Markers				
CEA (0-5 ng/mL)	21.3 ± 5.2	20.9 ± 4.8	2.6 ± 1.8	15.9 ± 3.8
CA199 (0-37U/mL)	132.6 ± 16.4	135.2 ± 18.6	16.6 ± 7.6	96.4 ± 11.4
NSE (0-16.3 μg/L)	57.1 ± 12.6	58.2 ± 13.4	7.1 ± 2.8	49.6 ± 13.0
CgA (20-100 ng/mL)	316.8 ± 28.2	322.2 ± 32.6	62.6 ± 16.2	284.2 ± 29.4
PP (10-100 pg/mL)	296.6 ± 16.8	291.4 ± 16.6	43.4 ± 8.6	262.6 ± 13.8
Race/Ethnicity, n (%)				
Han Chinese	16 (100%)	19 (100%)	10 (100%)	45 (100%)

Data presented as mean ± SD or n (%); *KRAS*-G12C Mutated PNETs: Patients with confirmed *KRAS*-G12C mutations; Wild-type *KRAS* PNETs: Patients without *KRAS* mutations; Healthy Controls: Volunteers with no history of pancreatic disease; BMI: Body mass index; PNETs: Pancreatic neuroendocrine tumors; NSE: Neuronspecific Enolase; CgA: Chromogranin A; PP: Pancreatic polypeptide.

#### Organoid models culture

Fresh KRAS^G12C^ mutant and wild-type PNET tissues were dissociated using collagenase IV (1 mg/mL, Sigma-Aldrich, MO, USA) and filtered through a 70 μm strainer. Cells were embedded in Matrigel (Corning, NY, USA) and cultured in Advanced DMEM/F12 medium supplemented with 10% FBS, 1× GlutaMAX, 10 mM HEPES, 100 U/mL penicillin-streptomycin, 50 ng/mL EGF, 100 ng/mL Noggin, and 500 ng/mL R-spondin1. Medium was refreshed every 3 days. Organoids were exposed to 100 μM CoCl_2_ (KA003, Cobiotech, China) for 24 h or cultured in a 1% O_2_ chamber (Thermo Fisher, MA, USA) for 48 h. For KRAS inhibition, 10 μM Sotorasib (MedChem Express, NJ, USA) was added 2 h prior to hypoxia induction.

### Method details

#### Immunohistochemistry (IHC)

Tumor tissues from 16 KRAS^G12C^-mutated PNETs and 19 wild-type KRAS controls were formalin fixed, paraffin-embedded, and sectioned at 4 μm thickness. Deparaffinized sections were rehydrated through graded ethanol and subjected to antigen retrieval using citrate pH 6.0 buffer. Endogenous peroxidase activity was blocked with 3% hydrogen peroxide for 15 min. Sections were incubated overnight at 4°C with primary antibodies targeting HLA-DR^+^ (1:200, Abcam, China), Tregs (CD4^+^, CD25^+^, FoxP3^+^), MDSCs (CD11b^+^, CD33^+^), or CD8^+^ (KB0001, Cobiotech, China). After washing, slides were incubated with HRP-conjugated secondary antibodies (Vector Laboratories) for 1 h at room temperature. Chromogenic detection was performed using a Vector NovaRED substrate kit (SK-4800, Vector, China), followed by counterstaining with hematoxylin. All slides were examined randomly by two independent pathologists, and IHC outcomes were determined by stained positive cell counts. Positive cells were counted in 0.02 mm^2^ windows and normalized to cells/mm^2^. Fluorescence intensity and integrated optical density (IOD) were analyzed using Image Plus 6.0 software, with background signals subtracted.

#### Flow cytometry

The study cohort comprised 16 patients with KRAS^G12C^ mutations PNETs, 19 patients with wild-type KRAS PNETs, and 10 healthy volunteers from a medical examination center as normal controls. Fresh blood samples were collected from all participants. Single-cell suspensions from fresh blood samples were stained with fluorochrome-conjugated antibodies targeting immune cell markers, including Tregs (CD4^+^, CD25^+^ and FoxP3^+^), activated T cells (HLA-DR^+^), cytotoxic T cells (CD8^+^), and MDSCs (CD11b^+^, CD14^+^, CD15^+^ and CD33^+^). Samples were analyzed using a BD FACSCanto II flow cytometer. Data were processed with FlowJo v10.8 software to quantify immune cell populations.

#### Structural analysis

PyMOL software (v3.1.0) was used to model interactions between the T cell specific antibody (PDB ID: 8VR9) and the HIF-1α-KRAS peptide conjugate. Key residues CYS-12 were highlighted to analyze covalent bond formation. Use the select command to highlight key amino acid residues such as CYS-12, GLU-63, TYR-159, GLY-10, TYR-99, and TRP-147 with the show sticks command. Select key amino acid residues of the KRAS peptide VAL-9, GLY-10, ALA-11 and highlight them using the show sticks command. Utilize the wizard mutagenesis tool in PyMOL to simulate the covalent bond formation between CYS-12 and HIF. Select the VH and VL domains of the T cell specific antibody and display the surface using the show surface command. Highlight key amino acid residues GLY-13, VAL-14, GLY-15, ASP, LYS-146, ASP-116 with the show sticks command. Save the image via File Export Image, choosing the appropriate format PNG and resolution.

#### Confocal microscopy analysis of KRAS protein expression

Tissue sections were permeabilized with 0.1% Triton X-100 for 10 min and blocked with 5% bovine serum albumin (BSA) for 1 h. Primary antibodies against KRAS, HIF-1α/β, HIF-2α(1:200, ab180772, Abcam, MA, USA) and DAPI (1:1000, D9542, Sigma-Aldrich, MO, USA) were applied overnight at 4°C. Secondary antibodies conjugated with Alexa Fluor 488 (green) or 594 (red) (Abcam, MA, USA) were incubated for 1 h at room temperature. Fluorescence images were captured using a Leica TCS SP8 confocal microscope with a 40× oil-immersion objective. Z-stacks were acquired at 0.5 μm intervals to ensure full cellular coverage. Image Plus 6.0 software was used to quantify fluorescence intensity by defining regions of interest around nuclei DAPI-positive and subtracting background signals. Optical density per cell was calculated and normalized to control samples.

#### qPCR analysis

Total RNA was isolated from frozen KRAS^G12C^ mutant and wild-type tumor tissues using TRIzol reagent. RNA purity and concentration were measured via NanoDrop 2000 (A260/A280 > 1.8). cDNA was synthesized from 1 μg DNase I-treated RNA using PrimeScript RT Kit (Bio-Rad, Hercules, CA, USA). Gene-specific primers for *HIF1A, ARNT* (encoding HIF-1β), *EPAS1* (encoding HIF-2α), and *GAPDH* (below Table) were used. qPCR was performed with SYBR Green Master Mix (Bio-Rad, CA, USA) on a CFX96 real-time system (Bio-Rad, CA, USA) under the following conditions: 95°C for 5 min, followed by 40 cycles of 95°C for 10 s, 60°C for 30 s, and 72°C for 20 s. Relative mRNA expression was calculated using the ΔΔCT method.

**Table:** Primer sequences for qPCR

Gene	Forward Primer (5′→3′)	Reverse Primer (5′→3′)
*HIF1A*	CAGTTACGGTGGACAGCCTC	AGTTGGTGGCAGTGGTAGTGG
*ARNT*	GAGGACCTGGAGATGGTGCT	TCCAGGTGTCCTTCCTCACC
*EPAS1*	TCTCCATCCCAGTGCTTTCC	AGCGGTGTGATGATGGTGAT
*GAPDH*	GGAGCGAGATCCCTCCAAAAT	GGCTGTTGTCATACTTCTCATGG

#### Western blotting assay

Proteins were extracted from KRAS^G12C^ mutant and wild-type tumor tissues using RIPA lysis buffer (#89900, Thermo Fisher Scientific, MA, USA) supplemented with protease and phosphatase inhibitors (P1006, Beyotime, Shanghai, China). Protein concentrations were quantified using a BCA Protein Assay Kit (Bio-Rad, CA, USA), following the manufacturer’s protocol. Equal amounts of protein (30 μg per lane) were separated by 10% SDS-PAGE and transferred to PVDF membranes (Millipore, MA, USA) using a semi-dry transfer system (Bio-Rad, CA, USA) at 15 V for 45 min. Membranes were blocked with 5% non-fat milk in TBST (Tris-buffered saline with 0.1% Tween 20) for 1 h at room temperature and then incubated overnight at 4°C with primary antibodies against HIF-1α (1:1000, ab180772, Abcam, MA, USA), HIF-1β (1:1000, ab2185, Abcam), HIF-2α (1:1000, ab199, Abcam), and β-tubulin (1:5000, ab6046, Abcam) as a loading control. After washing, membranes were incubated with HRP-conjugated secondary antibodies (1:5000, Cell Signaling Technology, MA, USA) for 1 h at room temperature. Protein bands were visualized using an ECL Prime Western Blotting Detection Kit (Bio-Rad, CA, USA) and imaged on a ChemiDoc XRS+ System (Bio-Rad, CA, USA). Band intensities were quantified using Image Lab 6.0 software (Bio-Rad, CA, USA) and normalized to β-tubulin expression.

#### Co-immunoprecipitation (CoIP)

For CoIP lysates 500 μg were pre-cleared with Protein A/G beads (Bio-Rad, CA, USA), then incubated with 2 μg anti-KRAS^G12C^ or anti-HIF-1α antibodies overnight at 4°C. Immune complexes were captured with Protein A/G beads, washed, and eluted in Laemmli buffer. Eluates were analyzed by Western blot as above. Organoid lysates treated with tumor-promoting factors 100 μM CoCl_2_ were processed similarly to assess KRAS^G12C^, HIF-1α, and HIF-1β co-expression. Band intensities were quantified using Image Lab 6.0 (Bio-Rad, CA, USA) and normalized to β-tubulin.

#### Laser micro-irradiation and live imaging

*KRAS*^G12C^ mutant organoids were transfected with GFP-tagged HIF-1α and SDH plasmid (#87230 Addgene, Cambridge, MA, USA) and stained with Hoechst 33342 (Thermo Fisher, Waltham, MA, USA). Localized HIF-1α sensory state stimulation was induced using a 405 nm multiphoton laser (50 mW, 1 Hz pulse) on a Leica TCS SP8 confocal microscope (5 μm spot diameter). GFP-KRAS^G12C^ recruitment to damage sites was recorded every 10 s for 10 min. Fluorescence intensity was quantified using ImageJ, and kinetic data were analyzed with LAS X software (Leica, Wetzlar, Germany).

### Quantification and statistical analysis

GraphPad Prism 9.0 was employed for data visualization and statistical testing. Group comparisons used Student’s *t* test or one-way ANOVA with Tukey’s post hoc test. Significance was defined as *p* < 0.05. Data are presented as mean ± SD.
