# Supplementary material for: Hypoxic-immune axis orchestrates metastatic dissemination via HIF isoform imbalance in pancreatic neuroendocrine tumors
Source: iScience. 2025 Dec 9;29(1):114340. doi: 10.1016/j.isci.2025.114340 (PMC12794431; doi:10.1016/j.isci.2025.114340)
Supplement: Document S1. Figure S1 [file mmc1.pdf]

iScience, Volume *29*

Supplemental information

Hypoxic-immune axis orchestrates metastatic dissemination via HIF isoform imbalance in pancreatic neuroendocrine tumors

Jianli Lin, Yi Lin, Min Cai, Yaoqi Chen, Xiafang Lin, Lizhi Li, Jianlin Lai, Huping Huang, Jinxin Li, Qinwen Liu, Qinyu Liu, Yinghua Luo, Xin Chen, and Jinsheng Liu

Supplementary Figure S-1

This set of supplementary data provides critical multi-level validation of the proposed hypoxic-immune axis in $KRAS^{G12C}$-mutant pancreatic neuroendocrine tumors (PNETs). Figure S-1A and S-1B first establish the clinical significance, demonstrating that *KRAS*-G12C mutation is strongly associated with aggressive disease, characterized by significantly worse progression-free survival and a higher incidence of synchronous metastasis. Figure S-1C delivers direct functional evidence, showing that pharmacological inhibition of either *HIF1A* or mutant *KRAS* itself suppresses the invasive capacity of patient-derived organoids in vitro and metastatic burden in vivo. The prognostic independence of this molecular signature is further confirmed by multivariable Cox analysis in Figure S-1D. Finally, Figure S-1E elucidates a key mechanistic link: targeted inhibition of $KRAS^{G12C}$ downregulates HIF-1α/β protein levels and reverses the associated immunosuppressive secretome. Collectively, these data bridge clinical observation with mechanistic insight, solidifying the HIF isoform imbalance as a central, therapeutically actionable driver of metastasis in this molecularly defined PNET subtype.

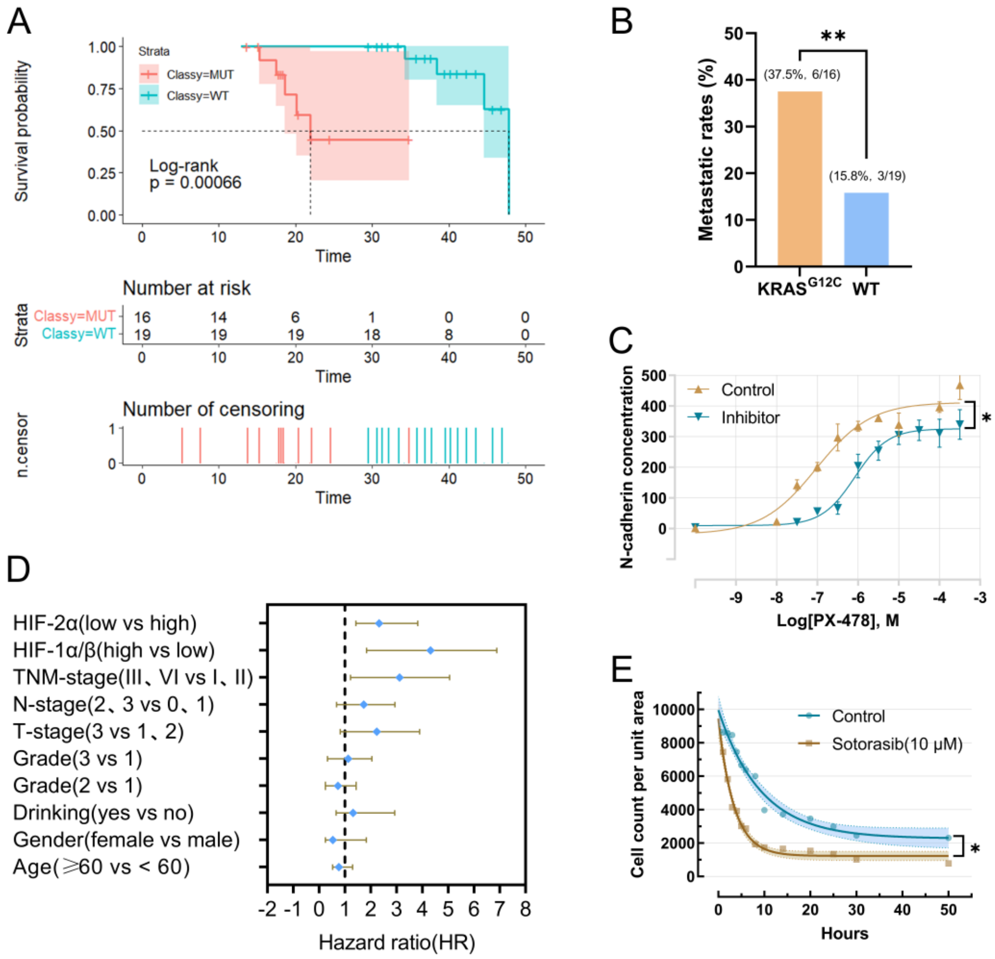

Figure S-1 Multi-level validation of the proposed hypoxic-immune axis in KRAS^{G12C}-mutant PNETs.

A. Kaplan-Meier analysis of progression-free survival (PFS) in patients with PNETs. Patients harboring *KRAS*-G12C mutations exhibit significantly shorter median PFS (18.2 months) compared to those with wild-type *KRAS* (38.5 months; log-rank, $p < 0.001$).

B. Bar graph showing the incidence of synchronous metastasis at diagnosis. The *KRAS*-G12C mutant group (6/16, 37.5%) demonstrates a higher metastatic rate than the wild-type *KRAS* group (3/19, 15.8%).

C. In vitro functional validation of the HIF–KRAS^{G12C} axis. Invasion assay using patient-derived organoids (PDOs) treated with HIF-1α inhibitor PX-478 (20 μM). Results showed that inhibitors significantly reduced organoid invasion and suppressed the expression of EMT markers N-cadherin ($p< 0.05$).

D. Forest plot from multivariable Cox regression analysis confirming that KRAS^{G12C} mutation and HIF-1α/β-high / HIF-2α-low signature are independent prognostic factors for poorer PFS, after adjustment for age, tumor grade, and stage.

E. Immunoblot and cytokine analysis of KRAS^{G12C}-mutant PDOs treated with sotorasib (10 μM). Treatment suppresses HIF-1α and HIF-1β protein levels and reverses the immunosuppressive secretome CXCL12, indicated by reduced count of tumor cell ($p< 0.05$).
